# A Case of Tracheal Adenoid Cystic Carcinoma in a Worker Exposed to Rubber Fumes

**DOI:** 10.1186/2052-4374-25-22

**Published:** 2013-10-17

**Authors:** Dohyung Kim, Yang-In Hwang, Sungyeul Choi, Chulyong Park, Naroo Lee, Eun-A Kim

**Affiliations:** 1 Occupational Safety and Health Research Institute, KOSHA, Incheon, Korea

**Keywords:** Adenoid cystic carcinoma, Occupational exposure, Rubber fumes, Curing

## Abstract

**Background:**

Primary tracheal tumors occur infrequently, accounting for less than 0.1% of all tumors. Adenoid cystic carcinoma (ACC) is the second most common type of malignancy of the trachea after squamous cell carcinoma (SCC). Little has been reported on the risk factors for tracheal ACC. The purpose of this study is to describe a case of tracheal ACC in a patient who had been exposed to rubber fumes, and to review the relationship between tracheal ACC and rubber fumes.

**Case report:**

A 48-year-old man who had been experiencing aggravation of dyspnea for several months was diagnosed as having ACC of the trachea on the basis of a pathologic examination of a biopsy specimen obtained via laser microscopy-guided resection. The patient had been exposed to rubber fumes for 10 years at a tire manufacturing factory where he worked until ACC was diagnosed. His job involved preheating and changing rubber molds during the curing process.

**Conclusion:**

ACC of both the trachea and the salivary glands show very similar patterns with regard to histopathology and epidemiology and are therefore assumed to have a common etiology. Rubber manufacturing is an occupational risk factor for the development of salivary gland tumors. Further, rubber fumes have been reported to be mutagenic. The exposure level to rubber fumes during the curing process at the patient’s workplace was estimated to be close to or higher than British Occupational Exposure Limits. Therefore, tracheal ACC in this case might have been influenced by occupational exposure to rubber fumes.

## Background

The incidence of primary tracheal carcinoma is 0.10 to 0.26 per 100,000 persons [1-5], accounting for 0.1–0.4% of total malignancy [6]. Adenoid cystic carcinoma (ACC) is the second most common type of primary tracheal tumors after squamous cell carcinoma (SCC) [7].

Due to the low incidence rate, little has been reported on the risk factors for tracheal ACC [8,9]. The only risk factors that have been investigated with regard to tracheal ACC are age, sex, and smoking status. Tracheal ACC has been found to develop more frequently in the fourth and fifth decades of life with no gender preference. Further, smoking status was not found to be associated with the development of tracheal ACC [6,10,11].

Rubber fumes consist of respirable dust generated during the compounding or curing processes of tire manufacturing. Although the specific carcinogen(s) have not yet been identified, several reports have described the mutagenic potential of rubber fumes [12-14]. The purpose of this study is to describe a case of tracheal ACC in a patient who had been exposed to rubber fumes while performing rubber curing in a tire manufacturing factory and to review the relationship between ACC development and exposure to rubber fumes.

## Case presentation

### Clinical course

A 48-year-old man first visited an otolaryngology clinic in May 2005 because of exertional dyspnea. His symptoms persisted and deteriorated over several months, and he was found to have a tracheal tumor. At this point, he was transferred to a university hospital. The patient did not have hypertension, diabetes, tuberculosis, viral hepatitis, or HIV infection. Several years earlier, he had undergone an operation for appendicitis. He did not have any known drug allergies. His family did not have a history of respiratory cancer. The patient reported that he had smoked approximately 4 cigarettes a day since he was 23 years old and had drunk 50 mL of Korean distilled spirits per day since he was 20 years old. He had no dyspepsia or resting dyspnea. Visual inspection of the larynx did not reveal any remarkable findings. The Epstein-Barr virus (EBV) antigen/antibody status was not available from the medical records.

Computed tomography (CT) of the patient’s neck, conducted on May, 2005, revealed a 3.5-cm polypoid soft-tissue tumor at the inner side of the right posterior wall of the subglottic region, extending to the outer side. The tumor blocked the inner cavity of the trachea. Laser microsurgery was conducted for partial excision of the tumor and frozen section biopsy. The final diagnosis on the basis of the frozen biopsy was tracheal ACC, with no extra-tracheal metastasis. Adjuvant radiation therapy was administered twice during next year. A follow-up medical examination was performed 5 years later, during which, chest radiography revealed multiple metastatic nodules in both lung fields. The patient subsequently received palliative chemotherapy.

### Occupational history

**Figure 1:**
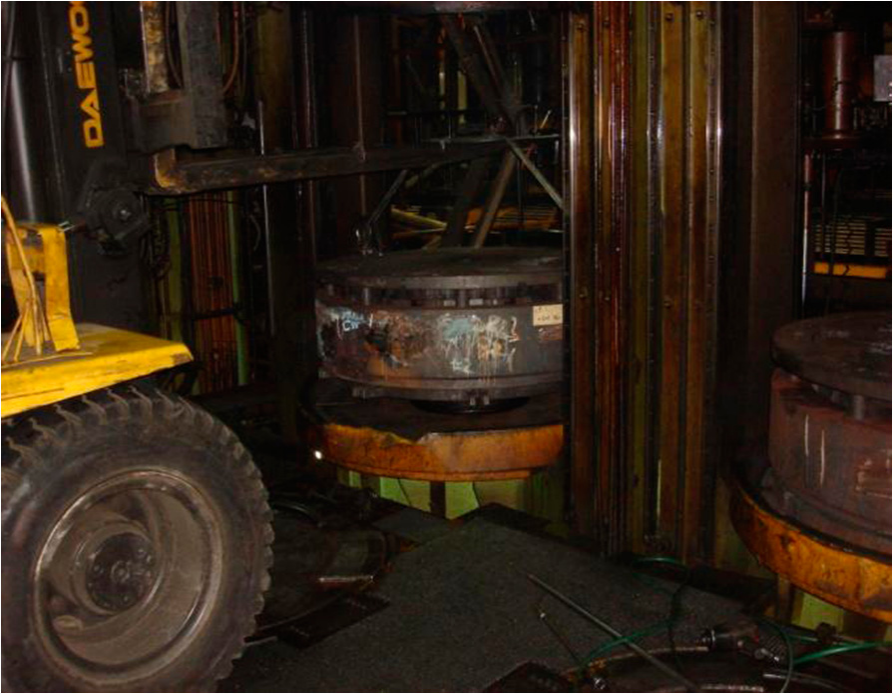
A worker is transferring and placing a mold by using a forklift.

**Figure 2:**
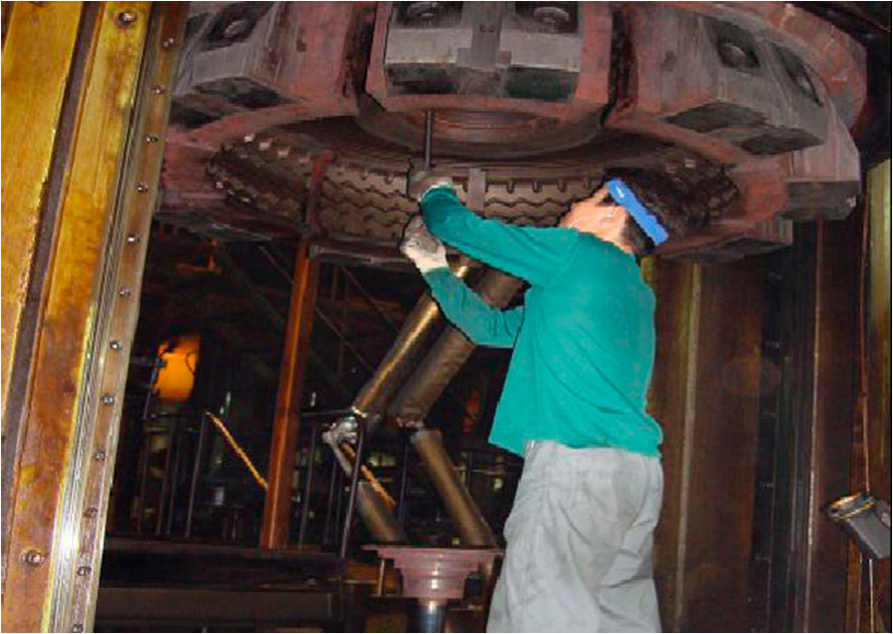
A worker is assembling a mold without any respiratory protective equipment.

The patient was discharged from the army after 3 years of service (1977–1980) and worked in rice farming (1981–1994). In 1995, he entered tire manufacturing, and was assigned to the curing process, where he worked for 10 years, until 2005. The curing process involved the use of 72 vulcanizers (installed with 144 molds). His main duty was preheating and changing molds and bladders. Groups of 3 workers were required to perform the mold-changing job. The workers moved the molds and bladders by using a forklift after the molds had been used several times or if the size of the tire being made needed to be changed (Figures 1 and 2). Molds were changed at a frequency of 3 to 5 times a day, and each change required approximately 1 to 2 hours. Preheating the mold required a high temperature of 180°C to 190°C for 4 to 6 hours. The patient was occasionally asked to clean the molds using an airbrush and apply anti-rust agents afterwards.

Available environmental measurement data provided by the company included information from 1997 to 2005, but information for 1999 was missing. The risk factor measured by the company was total dust until 2002. The company then changed to measuring rubber fumes (Table 1). During this period, the total dust and rubber fumes were measured to range from 0.03 to 0.91 mg/m^3^ and 0.03 to 0.57 mg/m^3^, respectively. The exposure level to total dust was less than 10% of the Occupational Exposure Limits (OEL) determined by the Korean Ministry of Employment and Labor [15]. The exposure level to rubber fumes was close to 95% of the British OEL (0.60 mg/m^3^) [16]. Other potential respiratory carcinogens that the patient might have been exposed to included N-nitrosamines and polycyclic aromatic hydrocarbons (PAHs) [17]. N-nitrosamines had not been measured until 2000 and had always been less than the detection limit. PAHs were measured to range from 1.4 to 22.6 μg/m^3^, which was less than 10% of the Korean OEL (200 μg/m^3^) [15]. Temperatures from 1998 to 2001 exceeded the limits for medium-grade labor intensity (75% of working, 25% of rest, 28°C) [15].

**Table 1:** Levels of chemicals in the work environment during the rubber curing process

	**OEL (TWA)**	1997^*^	**1998**	**2000**	**2001**	**2002**	**2003**	**2004**	**2005**
Total dust	10 mg/m^3^	NA	0.38 –0.64	0.09–0.91	0.03–0.31	NA	NA	NA	NA
Rubber fumes^†^	None^‡^	NA	NA	NA	NA	0.12–0.31	0.27–0.57	0.05–0.08	0.03–0.30
PAHs	200 μg/m^3^	NA	ND	3.8–11.6	1.4–22.6	3.2–4.4	NA	NA	NA
SO_2_	2 ppm	ND	NA	NA	NA	NA	0.04–0.10	0.04–0.09	0.01–0.08
N-Nitrosamine	None	NA	NA	NA	NA	NA	ND	ND	ND
1,3-Butadiene	10 ppm	NA	NA	NA	NA	ND	ND	ND	ND
Heat	28°C	NA	32	29.3	30.2	NA	23.0	27.1	26.9

NA: Not available.

ND: Non-detectable.

OEL: Occupational exposure limit.

TWA: Time weighted average.

PAHs: Polycyclic aromatic hydrocarbons.

*Data not available for 1995, 1996, and 1999.

^†^Measurement unit is mg/m^3^.

^‡^The Korean OEL is not defined, but the British OEL is 0.60 mg/m^3^[16].

There was a possibility of exposure to PAHs vaporized from anti-rust agents applied to molds due to the high temperature. In addition, cleaning the molds with an airbrush might have generated dust from pipe insulation materials. We analyzed a sample of those materials for the presence of asbestos and PAHs. The insulation material was found to contain glass fiber. Major components of the anti-rust agents were found to be mineral oil (CAS NO. 64742-55-8, 90%) and barium sulfate (CAS No. 25619-56-1, 7%); however, when these chemicals were exposed to temperatures similar to those used during the mold preheating process (180‒195°C ), PAHs were not detected, as assayed by the gas chromatography–mass spectrometry (GC-MSD) analytical method.

## Conclusion

Tracheal ACC is known to originate from the salivary glands of the trachea [6]. Therefore, it seems natural that tracheal ACC resembles salivary gland ACC of the head and neck (HNACC) in many ways. The histological similarity of tracheal ACC and HNACC has been thoroughly described [8]. Both of them consist of epithelial cells in cribriform, tubular, and solid growth patterns with hyaline or mucoid material-filled extracellular matrix, and show differentiation characteristics such as ductal-lining or myoepitheilal cells [9,18]. These two different cancers are thought to have very similar epidemiological characteristics with regard to age, sex, and smoking status, as mentioned earlier in the introduction [18].

These similarities indicate that tracheal ACC and HNACC may have common risk factors. Because HNACC is one of many subtypes of salivary gland tumors of the head and neck, risk factors for such a specific subtype are not well-known [18]. Therefore, it seems reasonable to assume the general risk factors of salivary gland tumors to be the same as those of HNACC. The general risk factors of salivary gland tumors are human immunodeficiency virus infection, Epstein-Barr virus infection, and ionizing radiation. It has been shown that workers from a variety of industries have an increased incidence of salivary gland tumors. These include rubber manufacturing, hairdressing, and industries with exposure to nickel compounds [18-20].

Mancuso *et al.*[21] suggested that nitroso compounds caused salivary gland tumors in laboratory mice, and the presence of nitroso compounds in rubbers could be an explanation for higher incidence of salivary gland cancer in rubber industrial workers. Vermeulen *et al.*[14] used *Salmonella typhimurium* strains, which overproduce specific enzymes such as nitroreductase and acetyltransferase in the presence of mutagenic material, and proposed that some components of rubber fumes may have mutagenic potential, and these may include aromatic amines. Kim B *et al.*[22] reported that once the nanoparticles in rubber fumes are deposited in the human body, they can easily translocate to other tissues and have a high toxicological effect due to their small size.

The present case was potentially exposed to carcinogens including PAHs and N-nitrosamines. PAHs are generated by incomplete combustion of organic material and are known to increase the risks of lung cancer and skin cancer [23]. N-nitrosamines are formed in the curing process when chemicals such as tetramethylthiuram disulfide, zinc diethyldithiocarbamate, or morpholino mercaptobenzothiazole are used [17]. N-nitrosamines are considered to be carcinogenic to humans on the basis of many experimental animal studies [24]. Among these carcinogens, it seems that the patient had been significantly exposed only to rubber fumes. Through the working period, the concentrations of other carcinogens such as PAHs and N-nitrosamines were non-detectable or less than 10% of the Korean OEL or even not available (Table 1). However, the unavailable rubber fume concentrations during the first 6 years (1996–2001) could be estimated by using a regression equation proposed by *Lee et al*[13]. Indeed, the estimated maximal value for the concentration of rubber fumes in this period was close to or higher than the British OEL (0.60 mg/m^3^) [16] (Table 2).

**Table 2:** Maximal concentrations of rubber fumes during the rubber curing process as estimated by a regression equation^*^

	**1996**	**1997**	**1998**	**1999**	**2000**	**2001**
Total dust^†^	NA	NA	0.64	NA	0.91	0.31
Rubber fumes^‡^	NA	NA	0.57	NA	0.79	0.30

NA: Not available.

*y = 0.81x + 0.049 (x = total dust, y = concentration of rubber fumes) adapted from Lee *et al.*[13].

^†^Measured concentration in mg/m^3^.

^‡^Estimated concentration in mg/m^3^.

This study has some limitations. We aimed to describe a relationship between rubber fume exposure and the development of tracheal ACC. First, for the 10-year exposure period, information regarding the first 6 years was not available. This forced us to rely on estimations based on statements from the patient and measurements from a tire manufacturing company in a previous study. Second, we presumed that the risk factors for head and neck salivary gland tumors and tracheal ACC are similar, which needs more thoughtful consideration. Third, it is known that the chemical composition analysis of rubber fumes is difficult and that data about their harmful effects are scanty [22], so we cannot but explain a biological plausibility for their carcinogenesis insufficiently. Fourth, some information about other possible risk factors such as EBV was not available, so we could not evaluate its effect on this case.

Despite the above limitations, this study is the first case report that suggests a relationship between exposure to rubber fumes and the development of tracheal ACC. However, in order to definitively prove the relationship between rubber fumes and occupational cancer, further epidemiological and experimental studies are necessary.

In conclusion, tracheal ACC and salivary gland tumor of head and neck have very similar patterns with regard to their histopathology and epidemiology, and may therefore share a common etiology. Rubber manufacturing is considered to be a risk factor of salivary gland tumors, and rubber fumes have been reported to have mutagenic and toxicological properties. Exposure levels of rubber fumes for this case are estimated to have been close to or higher than the British OEL, but those of other potential carcinogens such as PAHs and N-nitrosamines are not. Although the patient was an ex-smoker, tracheal ACC is not known to be associated with a smoking habit. Besides, this patient was not concluded to have been exposed to other risk factors of salivary gland tumors such as HIV infection or ionizing radiation. Therefore, it is suggested that the development of tracheal ACC in this case may have been influenced by occupational exposure to rubber fumes.

## Consent

Written informed consent was obtained from the patient’s guardian/parent/next of kin for the publication of this report and any accompanying images.

## Competing interests

The authors declare that they have no competing interests.

## Authors’ contributions

KDH and KEA conceived and designed the study. KDH, CSY and HYI were involved in conduction of the study. LNR and HYI performed the analysis and estimation of the environmental assessment. PCY and KEA interpretation of data. KEA, KDH, PCY, LNR and HYI were involved in writing the manuscript. KBG and YSD performed the revision the manuscript. All authors read and approved the final manuscript.
